# Is mobile phone influence the status of pilot or the flight safety?

**DOI:** 10.1371/journal.pone.0283577

**Published:** 2023-03-24

**Authors:** Shouxi Zhu, Hongbin Gu

**Affiliations:** 1 College of Civil Aviation, Nanjing University of Aeronautics and Astronautics, Nanjing Jiangsu, China; 2 Flight College of Binzhou University, Binzhou, Shandong, China; King Khalid University, EGYPT

## Abstract

**Background:**

This study aimed to explore the adverse influences of mobile phone usage on pilots’ status, so as to improve flight safety.

**Methods:**

A questionnaire was designed, and a cluster random sampling method was adopted. Pilots of Shandong Airlines were investigated on the use of mobile phones. The data was analyzed by frequency statistics, linear regression and other statistical methods.

**Results:**

A total of 340 questionnaires were distributed and 317 were returned, 315 of which were valid. The results showed that 239 pilots (75.87%) used mobile phones as the main means of entertainment in their leisure time. There was a significant negative correlation between age of pilots and playing mobile games (*p*<0.01). There was a significant positive correlation between the length of phone usage on rest days and the length of phone usage before sleep and the 15 items of the scale (*p*<0.01), age, flight hours and position had a significant negative influence on the total score of the scale (*p*<0.01), while the length of mobile phone usage on rest days and before sleep had a significant positive influence on the total score of the scale (*p*<0.01). Among the above five independent variables, the length of time spent using mobile phones on rest days is the most influential factor on pilot status.

**Conclusions:**

Excessive use of mobile phones is very common among pilots, and it has become one of the key factors affecting the status of pilots. In any case, the longer the pilots use mobile phones, the greater the adverse impacts on their own status. At the same time, the longer pilots use mobile phones, the greater threat to flight safety.

## Introduction

According to the research report released by Strategy Analytics, as of June 2021, about 4 billion people in the world have smart phones. According to the 48th Statistical Report on the Development of China’s Internet, released by the China Internet Network Information Center (CNNIC), the total number of mobile phone users in China has reached 1.614 billion, and the average person has more than one mobile phone. Over the years, smart phones have already entered everyone’s life and changed our way of life and entertainment. People not only use mobile phones when sitting and standing, but also when walking [1]. As one of the most frequently used digital products, the use of mobile phones not only brings convenience to people’s travel, shopping and entertainment, but also threatens people’s health and brings a series of problems [2].

According to statistics, most people have the habit of using mobile phones at night or before going to bed, which will reduce the amount of sleep. At the same time, excessive use of mobile phones will cause brain excitement, resulting in insomnia [3]. A study by Ibrahim Nahla Khamis suggested that advance effects of mobile phone usage could lead to dependency problems and the dependency was correlated with poor subjective sleep quality, and sleep latency [4]. At the same time, Jing Wen He studied the impact of using mobile phones before controlling sleep. The results showed that restricting mobile phone use before bedtime for four weeks was effective in reducing sleep latency, increasing sleep duration, improving sleep quality, reducing pre sleep ambient, and improving positive effect and working memory [5]. Mobile phone addition is also a common problem in mobile phone usage. The phenomenon of mobile phone addition among university students is becoming more and more serious. In addition to causing sleep related problems, mobile phone addiction often brings a series of psychological problems, through a cross-sectional study of 973 comprehensive universities in eastern China, Meng Liu found that sleep distractions had a partial mediating role in the relationship between mobile phone addition and depression symptoms [6].

Excessive use of mobile phones can also cause headache, dizziness, visual fatigue and a series of physical discomfort. Al-Khlaiwi Thamir investigated 437 Saudis and found that among the Saudi population, problems caused by excessive use of mobile phones such as headache, sleep disorder, tension, fashion and dizziness are widespread [7]. At the same time, more studies believe that the occurrence of visual fatigue is directly related to the excessive use of mobile phones, which will lead to a series of eye symptoms such as dry eyes, astringent eyes, and swollen eyes [8,9].

The impact of excessive use of mobile phones on work can not be ignored, especially in driving. A cross-sectional study by Oluwaseun Adeyemi shows that about 88% of the Nigerian drivers were prone to mobile phone addition, and about 65% reported having a road accident that was related to their mobile phone use while driving [10] On the whole, in the relevant research on mobile phone use, there are relatively few studies on the impact of mobile phones on work. For airline pilots, mobile phones obviously also lead to poor physiological and psychological changes. Different from other work, flight requires higher safety [11], and the abuse of mobile phones may pose a potential threat to flight safety. Therefore, it is necessary to study the use of mobile phones by pilots.

## Method

### 2.1 Participants

In this study, the pilots of Shandong Airlines were taken as the research participants, and the method of cluster random sampling was adopted. 340 respondents were selected from October 2022 to November 2022 to issue questionnaires. 317 questionnaires were returned, including 315 valid ones. The return rate of the questionnaire was 93.2%, and the effective rate of the questionnaire was 92.6%. Inclusion criteria of participants: using smart phones, physical examination certificates are within the validity period, with communication ability, and have no previous psychological issues during the last 6 months.

After deliberation by the Ethics Review Committee of Binzhou University, the experimental design and scheme of this study fully considered the principles of safety and fairness, and its research content would not cause harm and risk to the subjects. All participants were adults, the recruitment of participants will be based on the principle of voluntary and informed consent. The research process and data will be anonymous, and the privacy of participants will not be disclosed. In this paper, the form of online survey was adopted. Before the survey begins, participants will be informed of the relevant information of the questionnaire and asked to confirm their informed consent. If participants do not agree, the questionnaire will automatically exit. Therefore, all participants signed the informed consent form.

### 2.2 Questionnaire design

At present, there is no scale for the influence of mobile phone on pilots’ status. Therefore, a questionnaire is designed in this paper. According to the characteristics of pilots’ work, we divide the questionnaire into three dimensions. Dimension 1: The influence of mobile phone usage on the body; Dimension 2: Mobile phone addiction; Dimension 3: The direct impact of mobile phone usage on flight work. In order to improve the reliability and validity of the questionnaire, we designed the questionnaire based on part of the Mobile Phone Addiction Index (MPAI) compiled by Professor Yongchi Liang of Hong Kong University and part of the visual fatigue scale compiled by Yanyan Lin [12].

The questionnaire is mainly divided into 2 parts. The first part is the basic information, including gender, position, age, average time of using mobile phones on rest days, average time of using mobile phones before sleep, and frequently used mobile apps.

The second part is the scale. There are 5 items for each dimension and 15 questions in total. The Likert five level scale is adopted. The specific contents of the scale are shown in Table 1.

**Table 1:** Questionnaire.

Dimension	Order number	Item	1 Completely non compliant, 2 Non compliant, 3 Neutral, 4 Compliant, 5 Completely compliant
Dimension 1: the influence of mobile phone usage on the body	A1	My neck or shoulder aches from using my mobile phone	1 2 3 4 5
	A2	I always want to blink when I use my mobile phone	1 2 3 4 5
	A3	Using mobile phone makes it difficult for me to sleep	1 2 3 4 5
	A4	I always want to rub my eyes when I use my mobile phone	1 2 3 4 5
	A5	Using mobile phone causes my fingers to ache	1 2 3 4 5
Dimension 2: mobile phone addiction	B1	I often forget time because I spend too much time on mobile phone when I have nothing to do in spare time	1 2 3 4 5
	B2	I feel uncomfortable without my mobile phone	1 2 3 4 5
	B3	I found that mobile phone usage wasted a lot of time	1 2 3 4 5
	B4	When binge-watching dramas or novels, the time spends on mobile phone is often uncontrollable	1 2 3 4 5
	B5	I told myself that I should not use my mobile phone too much in the future	1 2 3 4 5
Dimension 3: the direct impact of mobile phone usage on flight work	C1	No matter what the situation is, as long as the mobile phone is around, I will always pick up and use it	1 2 3 4 5
	C2	In important flight segments such as takeoff and landing, I will think about interesting things in mobile phone	1 2 3 4 5
	C3	I sometimes feel sleepy when I work because I spend too much time on mobile phone	1 2 3 4 5
	C4	One second before the flight, I was still using my mobile phone	1 2 3 4 5
	C5	In the flight phase of the route, I will think about the interesting things in mobile phone	1 2 3 4 5

### 2.3 Reliability and validity analysis

Because the scale is not mature, in order to check the reliability of the scale, Cronbach’s α is used to test the reliability of the scale. The results are shown in Table 2. It can be seen that Cronbach’s α of each dimension of the scale is above 0.8, which indicates that there is a high internal consistency among the items at all levels of the scale, and the reliability of the data is high, which can be further analyzed.

**Table 2:** Reliability analysis.

Dimension	Cronbach’s α	Number of items
1	0.934	5
2	0.873	5
3	0.914	5

Table 3 shows the results of KMO and Bartlett’s tests. It can be seen that the KMO is 0.907, greater than 0.8, and the approximate chi-square is 4729.465, *p*<0.01. This indicates that each item is not independent. The three dimensions are interrelated and suitable for factor analysis. The analysis results are shown in Table 4.

**Table 3:** KMO and Bartlett’s test.

Kaiser-Meyer-Olkin-Measure of Sampling Adequacy		0.907
Bartlett’s test of sphericity	Approximate chi-square	4729.465
	Df	105
	Sig	0.000

**Table 4:** Results of structure validity analysis.

Item	Factor load coefficient			Communality
	Factor 1	Factor 2	Factor 3	
My neck or shoulder aches from using my mobile phone	**0.817**	0.364	0.216	0.846
I always want to blink when I use my mobile phone	**0.875**	0.213	0.278	0.888
Using mobile phone makes it difficult for me to sleep	**0.611**	0.263	0.422	0.621
I always want to rub my eyes when I use my mobile phone	**0.826**	0.378	0.161	0.851
Using mobile phone causes my fingers to ache	**0.806**	0.328	0.23	0.81
No matter what the situation is, as long as the mobile phone is around, I will always pick up and use it	0.212	0.402	**0.794**	0.836
In important flight segments such as takeoff and landing, I will think about interesting things in mobile phone	0.369	0.21	**0.797**	0.815
I sometimes feel sleepy when I work because I spend too much time on mobile phone	0.443	0.518	**0.515**	0.73
One second before the flight, I was still using my mobile phone	0.615	-0.026	**0.653**	0.806
In the flight phase of the route, I will think about the interesting things in mobile phone	0.617	0.115	**0.657**	0.825
I often forget time because I spend too much time on mobile phone when I have nothing to do in spare time	0.29	**0.612**	0.532	0.742
I feel uncomfortable without my mobile phone	0.066	**0.435**	0.734	0.732
I found that mobile phone usage wasted a lot of time	0.367	**0.763**	0.2	0.757
When binge-watching dramas or novels, the time spends on mobile phone is often uncontrollable	0.499	**0.468**	0.469	0.688
I told myself that I should not use my mobile phone too much in the future	0.242	**0.805**	0.227	0.758
Characteristic root value (before rotation)	9.386	1.01	1.31	-
Variance interpretation rate% (before rotation)	62.58%	6.73%	8.73%	-
Cumulative variance interpretation rate% (before rotation)	62.58%	78.04%	71.31%	-
Characteristic root value (after rotation)	4.805	2.998	3.903	-
Variance interpretation rate% (after rotation)	32.03%	19.99%	26.02%	-
Cumulative variance interpretation rate% (after rotation)	32.03%	52.02%	78.04%	-
Underline indicates that the absolute value of load factor is greater than 0.4				

It can be seen from Table 4 that the corresponding commonality value of all items are higher than 0.4, which indicates that the information of items can be effectively extracted. The variance interpretation rate of the three factors is 32.03%, 19.99%, 26.02% respectively, and the cumulative variance interpretation rate after rotation is 78.04%>50%, which means that the information content of the items can be effectively extracted. From the perspective of the factors corresponding to each item, it is also consistent with the basic expectations. Factor 1 corresponds to the first dimension, factor 2 corresponds to the second dimension, and factor 3 corresponds to the third dimension. The structural validity of the scale is good. At the same time, when designing the scale, we consulted relevant aviation experts, and the content validity of the scale can also be guaranteed.

In summary, the data obtained from the questionnaire are reliable and can be analyzed accordingly.

### 2.4 Statistical data processing

After the questionnaires are collected, we used Excel2013 and SPSSAU data analysis platforms for data statistical analysis, and by frequency statistics, linear regression and other statistical methods to study the data.

## Results

### 3.1 Basic information of participants

Basic information of 315 participants is shown in Table 2. It can be seen from Table 5 that the participants are all male, mainly aged from 31 to 35, with 185 captains and 130 co pilots. They are the backbone for airlines.

**Table 5:** Basic information of participants.

Category	Option	N	Percentage(%)
Gender	Male	315	100.0
	Female	0	0.00
Age group	20 to 25 years old	37	11.75
	26 to 30 years old	36	11.43
	31 to 35 years old	146	46.35
	36 to 40 years old	28	8.89
	41 to 45 years old	29	9.21
	46 to 50 years old	36	11.43
	Age more than 50 years	3	0.95
Position	Captain	185	58.73
	Co pilot	130	41.27
Flight hours	Within 500 hours	27	8.57
	500 to 1000 hours	8	2.54
	100 to 3000 hours	13	4.13
	3000 to 5000 hours	33	10.48
	5000 to 10000 hours	103	32.70
	More than 10000 hours	131	41.59
Total		315	100.0

### 3.2 Basic information of Mobile phone usage

Table 6 shows information about participants’ use of mobile phones. It can be seen that 239 pilots use mobile phones as the main means of entertainment in their leisure time, accounting for 75.87%. From the time of using mobile phones, 66 pilots used mobile phones for an average of 2.5 to 3 hours every day on rest days, accounting for 20.95%, while 64 pilots used mobile phones for an average of more than 4.5 hours, accounting for 20.32%. 107 pilots used mobile phones for 1 to 1.5 hours before sleep, and 114 pilots have kept the above habit of using mobile phones for more than 2 years.

**Table 6:** Mobile phone usage information.

Question	Option	N	Percentage(%)
Average length of mobile phone usage on rest days	Within 1 hour	18	5.71
	1 to 1.5 hours	15	4.76
	1.5 hours to 2 hours	42	13.33
	2 to 2.5 hours	21	6.67
	2.5 hours to 3 hours	66	20.95
	3 hours to 3.5 hours	15	4.76
	3.5 to 4 hours	54	17.14
	4 to 4.5 hours	20	6.35
	More than 4.5 hours	64	20.32
Average length of mobile phone usage before sleep	Within 30 minutes	89	28.25
	30 minutes to 1 hour	85	26.98
	1 hour to 1.5 hours	107	33.97
	1.5 hours to 2 hours	12	3.81
	More than 2 hours	22	6.98
How long have you kept the above habit of using the mobile phones	3 months	43	13.65
	3 to 6 months	29	9.21
	Half a year to one year	44	13.97
	1 to 1.5 years	31	9.84
	1.5 to 2 years	54	17.14
	More than 2 years	114	36.19
Whether the mobile phone is used as the main means of entertainment in leisure time	No	76	24.13
	Yes	239	75.87
Total		315	100.0

### 3.3 Correlation analysis between age and mobile apps

News aggregation apps (such as Toutiao), instant messaging apps (such as WeChat), Short video apps (such as Tiktok), reading apps (such as tomato novel), online shopping apps (such as taobao), watching videos (such as movies, TV dramas, etc.), and playing mobile games (such as Honor of Kings) are the main types of mobile entertainment apps. The Pearson Correlation Coefficient between the age of pilots and mobile apps they use frequently is shown in Table 7.

**Table 7:** Pearson Correlation Coefficient between the age of pilots and their frequent use of mobile apps.

Frequent use of mobile apps	Age group
News aggregation apps (such as Toutiao)	0.041
Instant messaging apps (such as WeChat)	-0.059
Short video apps (such as Tiktok)	0.009
Reading apps (such as tomato novel)	0.087
Online shopping apps (such as taobao)	-0.024
Watching videos (such as movies, TV dramas, etc.)	-0.037
Playing mobile games (such as Honor of Kings)	-0.170**

*p<0.05

**p<0.01.

It can be seen from Table 7 that there is no correlation between the age of pilots and news aggregation apps, instant messaging apps, short video apps, reading apps, online shopping apps, video watching, etc. (*p*>0.05). The correlation coefficient between the age of pilots and playing mobile games is -0.170 (*p*<0.01), indicating that there is a significant negative correlation between the age of pilots and playing mobile games, the older the pilots are, the less they like mobile games.

### 3.4 Correlation analysis of mobile phone usage duration and physical state

We use correlation analysis to study the correlation between the "average length of mobile phone usage on rest days", the " average length of mobile phone usage before sleep" and "my neck or shoulder aches from using my mobile phone", "I always want to blink when I use my mobile phone", "using mobile phone makes it difficult for me to sleep", "I always want to rub my eyes when I use my mobile phone", "using mobile phone causes my fingers to ache". The Pearson Correlation Coefficient results are shown in Table 8.

**Table 8:** Pearson Correlation Coefficient between mobile phone usage duration and physical state.

Item	Average length of mobile phone usage on rest days	Average length of mobile phone usage before sleep
My neck or shoulder aches from using my mobile phone	0.330**	0.059
I always want to blink when I use my mobile phone	0.283**	0.169**
Using mobile phone makes it difficult for me to sleep	0.291**	0.173**
I always want to rub my eyes when I use my mobile phone	0.306**	0.161**
Using mobile phone causes my fingers to ache	0.313**	0.201**

*p<0.05

**p<0.01.

As shown in Table 8, the Pearson Correlation Coefficients between the "average length of mobile phone usage on rest days" and "my neck or shoulder aches from using my mobile phone", "I always want to blink when I use my mobile phone", "using mobile phone makes it difficult for me to sleep", "I always want to rub my eyes when I use my mobile phone", "using mobile phone causes my fingers to ache" were 0.330, 0.283, 0.291, 0.306, and 0.313 respectively, and there was a significant positive correlation between them (*p*<0.01).

The Pearson Correlation Coefficients between the "average length of mobile phone usage before sleep" and "I always want to blink when I use my mobile phone", "using mobile phone makes it difficult for me to sleep", "I always want to rub my eyes when I use my mobile phone", "using mobile phone causes my fingers to ache" were 0.169, 0.173, 0.161, 0.201 respectively, and there was a significant positive correlation between them (*p*<0.01). The Pearson Correlation Coefficient between the "average length of mobile phone usage before sleep" and "my neck or shoulder aches from using my mobile phone" is 0.059, and the *p* value is 0.298>0.05. Which indicates that there was no correlation between them.

### 3.5 Correlation analysis of mobile phone usage duration and mobile phone addiction

We use correlation analysis to study the correlation between the "average length of mobile phone usage on rest days", the " average length of mobile phone usage before sleep" and "I often forget time because I spend too much time on mobile phone when I have nothing to do in spare time", "I feel uncomfortable without my mobile phone", "I found that mobile phone usage wasted a lot of time", "when binge-watching dramas or novels the time spends on mobile phone is often uncontrollable", "I told myself that I should not use my mobile phone too much in the future". The Pearson Correlation Coefficient results are shown in Table 9.

**Table 9:** Pearson Correlation Coefficient between mobile phone usage duration and mobile phone addiction.

Item	Average length of mobile phone usage on rest days	Average length of mobile phone usage before sleep
I often forget time because I spend too much time on mobile phone when I have nothing to do in spare time	0.220**	0.151**
I feel uncomfortable without my mobile phone	0.295**	0.120*
I found that mobile phone usage wasted a lot of time	0.285**	0.013
When binge-watching dramas or novels, the time spends on mobile phone is often uncontrollable	0.256**	0.043
I told myself that I should not use my mobile phone too much in the future	0.320**	0.149**

*p<0.05

**p<0.01.

As shown in Table 9, the Pearson Correlation Coefficients between the "average length of mobile phone usage on rest days" and "I often forget time because I spend too much time on mobile phone when I have nothing to do in spare time", "I feel uncomfortable without my mobile phone", "I found that mobile phone usage wasted a lot of time", "when binge-watching dramas or novels the time spends on mobile phone is often uncontrollable", "I told myself that I should not use my mobile phone too much in the future" were 0.220, 0.295, 0.285, 0.256 and 0.320 respectively, and there was a significant positive correlation between them (*p*<0.01).

The Pearson Correlation Coefficients between the "average length of mobile phone usage before sleep" and "I often forget time because I spend too much time on mobile phone when I have nothing to do in spare time", "I told myself that I should not use my mobile phone too much in the future" were 0.151 and 0.149 respectively, and there is a significant positive correlation between them (*p*<0.01). The Pearson Correlation Coefficient between the "average length of mobile phone usage before sleep" and "I feel uncomfortable without my mobile phone" is 0.120, and there was a significant positive correlation between them (*p*<0.05).

The Pearson Correlation Coefficients between the "average length of mobile phone usage before sleep" and "I found that mobile phone usage wasted a lot of time", "when binge-watching dramas or novels, the time spends on mobile phone is often uncontrollable" were 0.013 and 0.043 respectively, and *p*>0.05. Therefore, there was no correlation between them.

### 3.6 Correlation analysis of mobile phone usage duration and flight work

We use correlation analysis to study the correlation between the "average length of mobile phone usage on rest days", the " average length of mobile phone usage before sleep" and "no matter what the situation is, as long as the mobile phone is around, I will always pick up and use it", "in important flight segments such as takeoff and landing, I will think about interesting things in mobile phone", "I sometimes feel sleepy when I work because I spend too much time on mobile phone", "one second before the flight, I was still using my mobile phone", "in the flight phase of the route, I will think about the interesting things in mobile phone". The Pearson Correlation Coefficient results are shown in Table 10.

**Table 10:** Pearson Correlation Coefficient between mobile phone usage duration and flight work.

Item	Average length of mobile phone usage on rest days	Average length of mobile phone usage before sleep
No matter what the situation is, as long as the mobile phone is around, I will always pick up and use it	0.279**	0.214**
In important flight segments such as takeoff and landing, I will think about interesting things in mobile phone	0.265**	0.109
I sometimes feel sleepy when I work because I spend too much time on mobile phone	0.260**	0.213**
One second before the flight, I was still using my mobile phone	0.226**	0.181**
In the flight phase of the route, I will think about the interesting things in mobile phone	0.244**	0.148**

*p<0.05

**p<0.01.

As shown in Table 10, the Pearson Correlation Coefficients between the "average length of mobile phone usage on rest days" and "no matter what the situation is, as long as the mobile phone is around, I will always pick up and use it", "in important flight segments such as takeoff and landing, I will think about interesting things in mobile phone", "I sometimes feel sleepy when I work because I spend too much time on mobile phone", "one second before the flight, I was still using my mobile phone", "in the flight phase of the route, I will think about the interesting things in mobile phone" were 0.279, 0.265, 0.260, 0.226 and 0.244 respectively, and there was a significant positive correlation between them (*p*<0.01).

The Pearson Correlation Coefficients between the "average length of mobile phone usage before sleep" and "no matter what the situation is, as long as the mobile phone is around, I will always pick up and use it", "I sometimes feel sleepy when I work because I spend too much time on mobile phone", "one second before the flight, I was still using my mobile phone", "in the flight phase of the route, I will think about the interesting things in mobile phone" were 0.214, 0.213, 0.181 and 0.148 respectively, and there was a significant positive correlation between them (*p*<0.01). The Pearson Correlation Coefficient between the "average length of mobile phone usage before sleep" and "In important flight segments such as takeoff and landing, I will think about interesting things in mobile phone" was 0.109, and *p*>0.05. Therefore, there was no correlation between them.

### 3.7 Regression analysis of pilots’ mobile phone usage

Take the score of the scale as the dependent variable, and take flight hours, age, position, average length of mobile phone usage on rest days, average length of mobile phone usage before sleep as the independent variables for linear regression analysis. The results are shown in Table 11.

**Table 11:** Regression analysis results.

Independent variable	Regression Weights *B*	Standardized Regression Weights *Beta*	*R* ^*2*^	Adjusted *R*^*2*^	*t*	*p*
Age	-2.277	-0.220	0.048	0.045	-3.991	0.000**
Flight hours	-1.464	-0.145	0.021	0.018	-2.599	0.009**
Position	-5.133	-0.167	0.028	0.025	-3.003	0.003**
Average length of mobile phone usage on rest days	2.283	0.371	0.138	0.135	7.070	0.000**
Average length of mobile phone usage before sleep	1.982	0.172	0.030	0.027	3.093	0.002**

*p<0.05

**p<0.01.

It can be seen from Table 11 that age, flight hours, position, average length of mobile phone usage on rest days, and average length of mobile phone usage before sleep all have significant influence on the total score of the scale (*p*<0.01). Among them, age, flight hours and position had a significant negative influence on the total score (*p*<0.01), while the average length of mobile phone usage on rest days and before sleep had a significant positive impact on the total score. The *R*^*2*^ of the regression model corresponding to the five independent variables is 0.048, 0.021, 0.028, 0.138 and 0.030 respectively. Taking the average length of mobile phone usage on rest days as the independent variable, *R*^*2*^ of the corresponding regression model is 0.138, that is the largest. Which means that the average length of mobile phone usage on rest days can explain the reason for 13.8% of the total score and is the most influential factor among the five independent variables.

## Discussion

Mobile phones have become an indispensable tool for work or entertainment in people’s lives, as well as for pilots. According to the questionnaire in this paper, 75.87% of the pilots believed that mobile phones were the main means of entertainment in leisure time. Among 315 participants, only 33 pilots used their mobile phones on the average within 1.5 hours on rest days, accounting for only 10.47%. 36.19% of pilots have kept the above habit of using mobile phones for more than 2 years. Since the outbreak of the COVID-19 in 2019, in order to ensure the normal operation of flights, many pilots are often isolated and closed on rest days. Pilots can only spend time in watching TV or mobile phones in isolated hotels, which leads to a very simple means of entertainment for pilots, and the excessive use of mobile phones has become inevitable.

Among the several types of mobile apps that pilots use daily, except for mobile game, other types of apps have no correlation with the age of pilots. There was a significant negative correlation between the age of pilots and mobile games (*p*<0.01). Because most mobile games are designed for young people, not only the pilots, but basically all people’s love of mobile game will decrease with the increase of age.

In this paper, the scale for the influence of mobile phone on pilots’ status is divided into 3 dimensions, 5 items for each dimension, 15 items in total. The correlation analysis was used to study the correlation between the "average length of mobile phone usage on rest days", the "average length of mobile phone usage before sleep" and these 15 items respectively. The results showed that the average length of mobile phone usage on rest days had a significant positive correlation with the above 15 items (*p*<0.01), which showed that the longer the use of mobile phones on rest days, the more obvious influences on the status of pilots, in particular, there was a significant positive correlation between the 5 items directly related to flight work (*p*<0.01), which indicated that excessive use of mobile phones would affect pilots’ thinking activities, reduce their situational awareness, and be extremely detrimental to flight safety. The "average length of mobile phone usage before sleep" showed a significant positive correlation with 10 of the above 15 items (*p*<0.01), a significant positive correlation with "I feel uncomfortable without my mobile phone" (*p*<0.05). There is no correlation between "average length of mobile phone usage before sleep" and the following four items: "my neck or shoulder aches from using my mobile phone", "I found that mobile phone usage wasted a lot of time", "when binge-watching dramas or novels, the time spends on mobile phone is often uncontrollable" and "in important flight segments such as takeoff and landing, I will think about interesting things in mobile phone".

Using mobile phones before sleep is not a unique habit of pilots. Many people have the habit of lying in bed and check mobile phones before falling asleep. Compared with using mobile phones in the daytime, using mobile phones while lying down has less pressure on the cervical spine, so it is less likely to cause neck or shoulder pain. Similarly, since the use of mobile phones before sleep is not a unique behavior of pilots, compared with the use of mobile phones on rest days, the use of mobile phones before sleep has a relatively less impact on pilots.

The results of regression analysis showed that age, flight hours, position, average length of mobile phone usage on rest days and average length of mobile phone usage before sleep had a significant influence on the total score of the scale (*p*<0.01). Among them, age, flight hours and position can be regarded as the same type of variables, because with the growth of age, flight hours will naturally increase, and position will also naturally promote. These there independent variables have a significant negative influence (*p*<0.01), which shows that with the growth of age and flight experience, the adverse influence of mobile phone usage will decrease, but because there are many factors that affect the status of pilots, Therefore, *R*^*2*^ of the above independent variable regression equation is small. The average length of mobile phone usage on rest days and before sleep had a significant positive influence on the total score of the scale (*p*<0.01), indicating that in any case, The longer pilots use mobile phones, the greater adverse influences on their own status, and the greater threat to flight safety. Among the five independent variables, the *R*^*2*^ of regression model corresponding to the time spent using mobile phones on rest days is 0.138, which is the biggest factor influences the total score of the scale. This paper studies the relationship between the use of mobile phones and the status of pilots through questionnaires. Although *R*^*2*^ is still small, as we all know, there are many factors which can influence the status of pilots, and the average length of mobile phone usage on rest days can explain 13.8% of the change in the total score, it shows that the use of mobile phones has become one of the key factors influence the status of pilots. Obviously, excessive use of mobile phones by pilots should attract the attention of aviation management departments, and the management of pilots mobile phone usage has become a new direction of efforts to improve flight safety and efficiency.

The participants in this study are all men and no women, which may cause some limitations. Due to the particularity of the aviation industry, there are very few female pilots. According to incomplete statistics, the proportion of female pilots in China is only about 1.34%, and the proportion of female airline pilots is less than 0.5%. In many Arab countries, there are no female pilots at all, so only male participants will not affect the reliability of this study. In further study, we will expand the number of participants and increase a certain number of female pilots, which may lead to better research conclusions.

## Supporting information

S1 File Data sets of the study. (XLS)

## References

[1] Bartel KA, Gradisar M, Williamson P. Protective and risk factors for adolescent sleep: A meta-analytic review. Sleep Med Rev. 2015;21:72–85. doi: 10.1016/j.smrv.2014.08.002 25444442

[2] Kubiszewski V, Fontaine R, Rusch E, Hazouard E. Association between electronic media use and sleep habits: an eight-day follow-up study. International Journal of Adolescence & Youth. 2014;19(3):395–407.

[3] Liese E, Van den Bulck Jan. Bedtime mobile phone use and sleep in adults. Soc Sci Med. 2016;148(1):93–101. doi: 10.1016/j.socscimed.2015.11.037 26688552

[4] Ibrahim NK, Baharoon BS, Banjar WF, Jar AA, Ashor RM, Aman AA, et al. Mobile Phone Addiction and Its Relationship to Sleep Quality and Academic Achievement of Medical Students at King Abdulaziz University, Jeddah, Saudi Arabia. Journal of Research in Health Sciences. 2018;18(3):e420. PMC6941644 30270211

[5] He JW, Tu ZH, Xiao L, Su T, Tang YX. Effect of restricting bedtime mobile phone use on sleep, arousal, mood, and working memory: A randomized pilot trial. Plos One. 2020;15(2):e228756. doi: 10.1371/journal.pone.0228756 32040492 PMC7010281

[6] Liu M, Lu C. Mobile phone addiction and depressive symptoms among Chinese University students: The mediating role of sleep disturbances and the moderating role of gender. Front Public Health. 2022;10:965135. doi: 10.3389/fpubh.2022.965135 36203678 PMC9531624

[7] Thamir A, A MS. Association of mobile phone radiation with fatigue, headache, dizziness, tension and sleep disturbance in Saudi population. Saudi Med J. 2004;25(6):732–7. 15195201

[8] Yujie M, Xiao S, Kelan Y, Xin W, Fangli F, Yaying W, et al. Comparison of the influence of light between circularly polarized and linearly polarized smartphones on dry eye symptoms and asthenopia. Clinical and translational science. 2021;15(4):994–1002. doi: 10.1111/cts.13218 34962062 PMC9010255

[9] Yunhong Z, Feng S, Yi Y, Jin Y, Wei L, Jingtian Q. The Evaluation and Testing on Visual Fatigue and Comfort Between Different Types of VR HMDS and Their Corresponding Pad or Phone. SID Symposium Digest of Technical Papers. 2021;52:62–65.

[10] Adeyemi O. The Association of Mobile Phone Addiction Proneness and Self-reported Road Accident in Oyo State, Nigeria. Journal of Technology in Behavioral Science. 2021;6:486–491.

[11] Tzelepi M, Tefas A. Graph Embedded Convolutional Neural Networks in Human Crowd Detection for Drone Flight Safety. IEEE Transactions on Emerging Topics in Computational Intelligence. 2019;5(2):191–204.

[12] Lin Y, Deng R, Li Z. A new valid and reliable questionnaire of asthenopia: development and evaluation. Chinese Journal of Ophthalmology. 2021;57(4):284–91. doi: 10.3760/cma.j.cn112142-20200701-00442 33832053

